# Tei Index and its Relation to Outcome of Critically Ill Children on Continuous Renal Replacement Therapy

**DOI:** 10.1007/s12098-023-04903-3

**Published:** 2023-12-22

**Authors:** Fatina I. Fadel, Ahmed M. Badr, Marwa M. Abdelkareem, Mohammad Samir, Mohammad Abdallah, Fatma Mohammad Atia, Yasmin M. Ramadan

**Affiliations:** Department of Pediatrics, Center of Pediatric Nephrology and Transplantation (CPNT), Cairo University, Cairo, Egypt

**Keywords:** Critically ill, Children, Renal replacement, Echocardiography, Tei index

## Abstract

**Objectives:**

To evaluate echocardiographic parameters, especially the Tei index as a predictor of outcome in critically ill children on continuous renal replacement therapy (CRRT).

**Methods:**

This cohort study included all critically ill patients admitted at the Pediatric intensive care unit (PICU) and underwent CRRT. Functional echocardiography and Pediatric Risk of Mortality Index (PRISM) III were used to evaluate the participants. Both the Tei index and the Vasoactive inotropic score (VIS) were estimated.

**Results:**

The study included 35 patients with an age range of 6 mo to 14 y. The Tei indexes, VIS, and PRISM III were reported as predictors of mortality with a sensitivity of 88%, 83%, and 94% and a specificity of 73%, 79%, and 89% respectively. In survivors, the mean Tei index score, median VIS, and mean PRISM values were 0.44 ± 0.1, 3.8 (0–40), and 12.06 ± 3.35, respectively. However, in non-survivors, the mean Tei index, median VIS, and mean PRISM score were 0.59 ± 0.16, 0.60 (0–342.5), and 22.94 ± 8.93, respectively.

**Conclusions:**

The Tei index could be used as a predictor for poor outcomes in children receiving CRRT. It is correlated to the PRISM score and VIS.

## Introduction

Acute kidney injury (AKI) affects about 30% of critically ill children in pediatric intensive care units (PICUs). Even though continuous renal replacement therapy (CRRT) is used to support kidney function in critically ill adults with severe AKI and/or multiple organ dysfunction syndrome, its use in the PICU is uncommon [1].

Children requiring CRRT have a higher mortality, which appears to be related to the underlying illness and fluid overload [2]. There is a knowledge gap regarding predictors of mortality in children receiving CRRT.

Scoring systems for mortality in PICU patients would predict prognosis. The Pediatric Risk of Mortality Index (PRISM), the Pediatric Index of Mortality (PIM), and Pediatric Logistic Organ Dysfunction (PELOD) are the most commonly used scoring systems [3].

Cardiac dysfunction is a risk factor that contributes to the development or worsening of AKI and it has been connected to a higher risk of mortality [4].

The Tei index or the Myocardial performance index (MPI) predicts global myocardial performance and allows for systolic and diastolic evaluation. Echocardiographic tissue Doppler is used to measure this index. Compared to other classical echocardiographic parameters, it is unaffected by volume status, systemic hypertension, and heart rate [5].

In this study, authors aimed to evaluate the Tei index in predicting the prognosis in children receiving CRRT.

## Material and Methods

This study was a prospective study, conducted in PICU at the University Hospital. The research was approved by authors’ institute’s ethical committee and was conducted in accordance with the Helsinki Declaration.

The study’s primary outcome was the effectiveness of Tei index as a predictor of mortality in children receiving CRRT.

Thirty-five critically ill children admitted to PICUs were enrolled. This number represented all patients undergoing CRRT in the PICUs between January 2020 and September 2021. All patients included had hemodynamic instability (hypotension and/or poor peripheral perfusion) that interfered with intermittent hemodialysis (IHD). Also, children who developed early cardiovascular collapse following initiation of IHD were considered intolerant and indicated for CRRT.

All these patients had renal impairment, with 18 (51.4%) having chronic kidney disease (CKD) and 17 (48.6%) having AKI.

The authors focused on patients’ age, weight, chronic disease history, cause of ICU admission, need for mechanical ventilation, onset, duration, indications for CRRT, anticoagulation during CRRT, and laboratory findings, including kidney function tests, electrolytes, blood gases, and sepsis profile.

Using GE Vivid E95, a single, well-trained cardiologist, assessed the heart via two-dimensional and tissue Doppler echocardiography. Left ventricular posterior wall thickness (LVPWT), interventricular septum thickness (IVST), fractional shortening (FS), and ejection fraction (EF) were measured using M-mode in the parasternal long-axis view.

Doppler blood flow velocities (peak E and A wave velocity) were obtained for the mitral and tricuspid valves using the apical view to detect diastolic function [6].

Tei index was calculated by the sum of isovolumic relaxation time and isovolumic contraction time divided by ventricular ejection time. To measure the LV Tei index, a continuous wave cursor line was positioned midway between the LV outflow tract and the anterior mitral leaflet, while for the RV Tei index, it was positioned at the lateral portion of the tricuspid annulus.

In young children, breath-holding cannot be utilized to counteract the effect of respiration on blood velocity, so three cardiac cycles were monitored, and the maximum velocity was selected. An average of the three Doppler measurements were taken to reduce observer variation.

Z score was used to normalize echocardiographic findings because of variations in age and body mass index and it was considered abnormal when the value was more than 2 Z score [7].

The PRISM III score, which is an index of overall sickness of critically ill children, was calculated at ICU admission and before starting CRRT. The PRISM III score (zero-74) is composed of 17 physiologic variables which are divided into 26 ranges. Physiologic variables were obtained in the first 12 h of ICU care, while laboratory variables were obtained in the period from 2 h before ICU admission through the first 12 h. A higher score equated to a worser prognosis [8] .

The maximum doses of the inotropes received were revised and the vasoactive inotropic score (VIS) was calculated [9].

Recovery of renal function, cure of underlying condition, or improved hemodynamics (normalization of heart rate, capillary refill, volume of peripheral pulsation, and blood pressure) permitting transfer to IHD were all criteria for successful CRRT cessation.

The Statistical Package for the Social Sciences (SPSS) version 26 was used to interpret data. For numerical data, the mean ± standard deviation (SD) or median (interquartile range) were used. For categorical data, the number (percent) was used. To compare quantitative variables, Mann-Whitney tests for non-parametric values and the t-test for parametric values were used. While, Chi square (χ2) test was used to compare the categorical data. Using the Spearman correlation coefficient, correlations between non-parametric quantitative variables were determined [10]. *P*-values below 0.05 were considered statistically significant.

The minimum convenient sample size was 25 patients as this number of patients would achieve a power of 80% considering the survival rate of 18 cases (72%), a difference of 25% in primary outcomes (Tei index, FS and PRISM score) between survivors and non-survivors, these calculations were performed using ClinCal online calculator.

## Results

The median age and weight of the patients studied, as well as the indications for CRRT are listed in Table 1. The mean PRISM III score prior to the start of the CRRT session was 17.34 ± 8.58, with a range of 5–38.

**Table 1:** The clinical characteristics of the cohort studied at the initiation of CRRT (n = 35)

Clinical characteristics		Median (IQR)	Range
Age (months)		72 (36–132)	6–168
Weight (kg)		20 (13–29)	6–50
Main indications of starting CRRT, n (%)			
Metabolic acidosis Volume overload Refractory hyperkalemia Uremic symptoms		23 (65.7) 19 (54.3) 11 (31.4) 3 (8.5)	

	Before CRRT	After CRRT	*P* value
Scores			
Glasgow coma scale, mean ± SD Normal pupillary response^a^ VIS, median (IQR)	9.34 ± 3.5 26 (74.3) 20 (0–342.5)	9.37 ± 4.6 23 (65.7) 15 (0–342.5)	0.95 0.01 0.22
Mechanical ventilation^a^	28 (80)	23 (65.7)	0.125
CRP, median (IQR)	48 (12–96)	48 (24–96)	0.17

*CRP* C-reactive protein, *CRRT* Continuous renal replacement therapy, *IQR* Inter quartile range, *SD* Standard deviation, *VIS* Vasoactive inotropic score

^a^Number (%)

The median time of CRRT initiation was 24 h (IQR = 12–72) while the duration of CRRT session ranged from 20 h to 72 h with mean 50 ± 19 SD.

The majority of cases were put on continuous veno-venous hemodiafiltration (CVVHDF) representing 85% of the total cases. While, continuous veno-venous hemofiltration (CVVH) represented 8.6%, continuous veno-venous hemodialysis (CVVHD) 2.9% and (slow continuous ultrafiltration) (SCUF) represented 2.9%. Heparin was used in almost all patients (97.1%) while saline flush was used in only one patient (2.9%).

The PRISM III score showed an inverse relationship with the outcome; the mean PRISM III scores for survivors and non-survivors were 12.06 ± 3.35 and 22.94 ± 8.93 with a highly significant *P* value <0.001.

According to authors’ institutional protocol, inotropic medication was initiated if the patient had hypotension, poor peripheral perfusion, or heart failure. The first-line inotropic medication in the current study were noradrenaline (57.1%, n = 20) and adrenaline (54.3%, n = 19). Other inotropic drugs were dobutamine (31.4%, n = 11), milrinone (14.3%, n = 5), dopamine (5.7%, n = 2), and vasopressin (2.9%, n = 1). Prior to CRRT, the median VIS was 20 (0–342.5), which did not significantly improve after CRRT discontinuation, reaching 15 (0–342.5). The median VIS was significantly (*p* <0.001) lower among survivors [3.8 (0–40)] compared with non-survivors [60 (0–342.5)].

In the patients studied, the mean FS was 28.37 ± 9.6, and 18 patients (51.84%) had impaired LV systolic function (FS <28%). Twelve subjects were found to have global ventricular dysfunction; five had a high Tei RV index Z score and seven had a high Tei LV index Z score (Table 2).

**Table 2:** Echocardiographic findings of the patients studied before the initiation of the CRRT session

	Range	Mean ± SD
IVST Z score^a^	0.23–0.9 -6.10–6.34	0.58 ± 0.14 0.35 (-2–1.9)
LVPWT Z score^a^	0.36–0.8 -2.96–5	0.54 ± 0.11 0.06 (-1.80–1.13)
Fraction shortening (FS) Z score^a^	15–57 -6.34–5.68	28.37 ± 9.6 -3.16 (-4.54– -0.66)
Ejection fraction Z score^a^	30–86 -5.51–7.5	55.03 ± 11.65 -0.89 (2.81–0.85)
M E/A Z score^a^	0.89–2.4 -2.15–0.38	1.45 ± 0.4 -0.98 (1.55– -0.44)
RV E/A Z score^a^	0.67–2.1 -2.06–0.8	1.45 ± 0.34 -0.49 (-0.84– -0.16)
End-systolic pulmonary artery pressure	20–60	29.89 ± 7.9
RV Tei index Z score^a^	0.31–0.96 -0.94–4.5	0.48 ± 0.15 0.28 (-0.15–1.32)
LV Tei index Z score^a^	0.29–0.92 -0.78–4.8	0.51 ± 0.15 0.64 (0.12–1.50)

*IVST* Interventricular septal thickness at end diastole, *LV* Left ventricle, *LVPWT* Left ventricular posterior wall thickness, *M* Mitral valve, *RV* Right ventricle

^a^Median and IQR; *E/A* The ratio of peak velocity blood flow in early diastole (the E wave) to peak velocity flow in late diastole caused by atrial contraction (the A wave)

In this study, the mean Tei index (RV and LV) in cases with AKI was 0.52 ± 0.2 and 0.53 ± 0.2 respectively, while in cases with CKD, it was 0.45 ± 0.1 and 0.49 ± 0.1 respectively, and there was no statistically significant difference between them (*p* = 0.17 and 0.46, respectively).

In this study, 71.4% (n = 25) of patients had their CRRT sessions successfully terminated compared to 29.6% (n = 10) who had their sessions terminated due to hypotension (n = 5, 14.3%), patient death (n = 3, 8.6%), or technical issues (n = 2, 5.7%).

The main components of the PRISM III score that differed significantly between survivors and non-survivors were blood pressure, consciousness level, kidney functions, and pupillary reaction (Table 3).

**Tabl﻿e 3:** Comparison between survivors and non-survivors regarding the PRISM III scores and echocardiographic findings before initiation of the CRRT session

Components of PRISM III scores	Survivors (n = 18) (Mean ± SD)	Non-survivors (n = 17) (Mean ± SD)	t^a^	*P* value
Blood pressure (mmHg)				
Systolic Diastolic	107.61 ± 42.1 62.83 ± 25.04	74.53 ± 26.02 43.94 ± 17.4	2.812 2.605	0.01 0.01
Glasgow coma scale	11.22 ± 2.6	7.35 ± 3.3	3.866	<0.001
PRISM III scores	12.06 ± 3.4	22.94 ± 8.9	4.723	<0.001

	N (%)	N (%)	X^2^^b^	
Fluid overload	8 (42.1)	11 (57.9)	1.45	0.23
VIS, median (IQR)	3.8 (0–40)	60 (0–342.5)	6.81	0.001
Abnormal pupillary reaction	1 (11.1)	8 (88.9)	7.88	0.01

Echocardiographic findings, median (IQR)				
IVST Z score	0.84 (-1.56–1.30)	0.14 (-2.31–2.3)	0.23^c^	0.82
LVPWT Z score	0.12 (-1.60–1.79)	-0.30 (-2.29–0.6)	1.17^c^	0.24
FS Z score	-1.45 (-4.50–0.04)	-3.95 (-4.6– -2.2)	1.73^c^	0.08
EF Z score	-0.07 (-2.2–0.85)	-2.20 (-3.52–0.36)	1.19^c^	0.24
M E/A Z score	-1.07 (-1.7– -0.4)	-0.80 (-1.35– -0.5)	0.46^c^	0.64
T E/A Z score	-0.48 (-1.26– -0.2)	-0.49 (-0.8– -0.17)	0.58^c^	0.56
ESPAP, mean ± SD	29.44 ± 9	30.35 ± 6.7	0.34	0.74
RV Tei index, mean ± SD Z score, median (IQR)	0.42 ± 0.1 0.07 (-0.34–0.54)	0.54 ± 0.17 0.61 (0.13–1.42)	2.50 2.41^c^	0.02 0.02
LV Tei index, mean ± SD Z score, median (IQR)	0.44 ± 0.1 0.21 (-0.16–0.87)	0.59 ± 0.16 1.36 (0.57–2.1)	3.43 2.97^c^	0.002 0.003

*CRRT* Continuous renal replacement therapy, *E/A* The ratio of peak velocity blood flow in early diastole (the E wave) to peak velocity flow in late diastole caused by atrial contraction (the A wave), *EF* Ejection fraction, *ESPAP* End-systolic pulmonary artery pressure, *FS* Fraction shortening, *IVST* Interventricular septal thickness, *LV* Left ventricle, *LVPWT* Left ventricle posterior wall thickness, *M* Mitral valve, *PRISM* *score* Pediatric risk of mortality score, *RV* Right ventricle, *T* Tricuspid valve, *VIS* Vasoactive inotropic score

^a^Student t-test

^b^Chi-square test (*FE* Fisher Exact test)

^c^Mann-Whitney U test (Median and IQR)

**Fig. 1:**
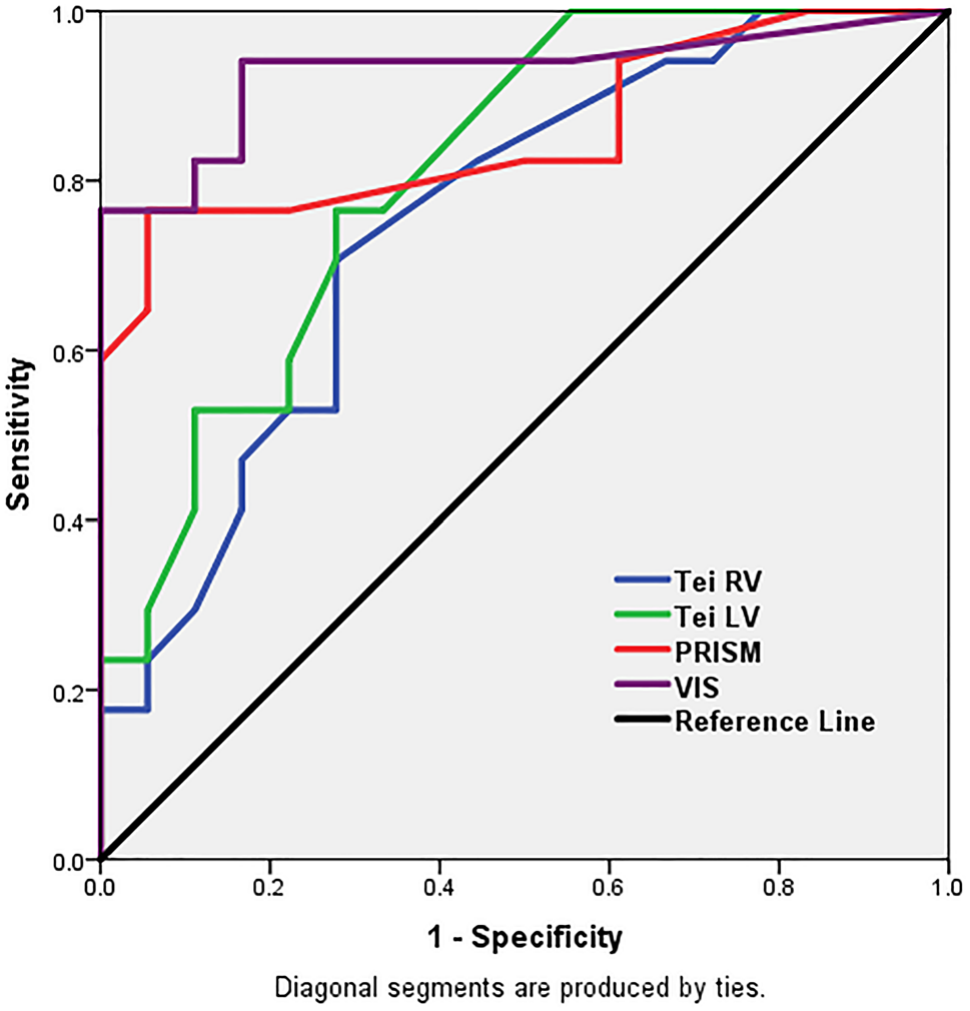
Receiver operating characteristic (ROC) curve for the mortality predictors among the studied cohort. *PRISM score* Pediatric risk of mortality score, *Tei LV* Tei score of left ventricle, *Tei RV* Tei score of right ventricle, *VIS* Vasoactive inotropic score

The LV and RV Tei index values, as well as their Z scores, were significantly inversely related to mortality (Fig. 1).

The authors correlated the Tei index of both RV and LV with other predictors of mortality and found that it was significantly correlated with PRISM III score and VIS (Table 4).

**Table 4:** Univariate correlations and multivariate analysis of the Tei index with other predictors of mortality

	Univariable correlation^a^	*P* value	Multivariable Odds ratio^b^	95% CI		*P* value
				Lower	Upper	
RV Tei index						
Age (years)	-0.007	0.48	0.002	-0.007	0.011	0.658
DBP (mmHg)	-0.183	0.150	-0.001	-0.048	0.032	0.689
SBP (mmHg)	-0.099	0.29	0.001	-0.06	0.071	0.862
PRISM III score	0.387	0.01	0.051	0.008	0.094	0.022
VIS	0.361	0.02	0.007	0.002	0.015	0.013
LV Tei index						
Age (years)	-0.143	0.21	0.002	-0.007	0.011	0.596
DBP (mmHg)	-0.229	0.093	-0.022	-0.062	0.014	0.254
SBP (mmHg)	-0.204	0.12	0.001	-0.007	0.008	0.341
PRISM III score	0.393	0.01	0.057	0.015	0.099	0.010
VIS	0.526	0.001	0.007	0.001	0.013	0.020

*CI* Confidence interval, *DBP* Diastolic blood pressure, *LV* Left ventricle, *PRISM score* Pediatric risk of mortality score, *RV* Right ventricle, *SBP* Systolic blood pressure, *VIS* Vasoactive inotropic score

^a^Spearman correlation was used

^b^t-test was used

In the present study, 53% (n = 9) of non-survivors had pericardial effusion compared to only 17% (n = 3) of survivors (*p* = 0.05). Pericardial effusion ranged in severity from 5 mild cases, 1 moderate case, and 3 severe cases among the non-surviving patients.

Estimated pulmonary artery pressure (ESPAP) ranged from 20 to 60 with mean 29.89 +/-7.88 SD, with no significant difference between survivors (mean = 29.44 mmHg ± 9 SD) and non survivors (mean = 30.35 mmHg ± 6.73 SD) with *P* value = 0.74.

C-reactive protein (CRP) level ranged from 6 to 196 with median 48 and IQR 24–96; it was significantly higher in non-survivors than in the survivors as the median CRP was 24 (12–48) mg/L in survivors and 56 (24–132) mg/L in non-survivors (*P* = 0.03) with no significant difference between CRP levels before and after CRRT (*p* value = 0.17) as shown in Table 1.

A patient who was mechanically ventilated had a poor prognosis; out of 18 survivors only 7 were mechanically ventilated whereas all 17 non-survivors were ventilated (*P* = 0.01).

Timing of CRRT initiation was inversely related to the patient’s outcome where the median time in the survivors was 12 (12–24) h and 24 (12–72) h in non-survivors (*p* = 0.05). The authors found that the time of onset of CRRT cannot predict mortality; AUC = 0.68 with 0.09 standard error, *P* = 0.07 CI (0.5–0.86).

Mortality predictive value was reported, and it was shown to be excellent for VIS, high for both PRISM III and the Tei LV index, and good for the Tei RV index (Fig. 1).

## Discussion

CRRT restores water and electrolytes and promotes renal function recovery. CRRT efficiently removes proinflammatory and anti-inflammatory cytokines, regulating the immune system [11]. However, CRRT has several drawbacks, including a long implementation time, the use of anticoagulation, the need for multiple venous accesses, the use of high volumes of replacement fluid, the immobility of patients receiving CRRT, and the type of patient with multiorgan system failure that increases mortality [12].

A bedside point-of-care echocardiography can provide real-time hemodynamic information by assessing cardiac function, loading conditions (preload and afterload) and cardiac output [13]. On the other hand, conventional echocardiographic parameters like left ventricular ejection fraction (LVEF) and left ventricular fractional shortening (LVFS) could miss early changes in cardiac function as they are affected by loading conditions and inotropic use [14]. The Tei index can detect early ventricular systolic and diastolic dysfunction and it is not affected by ventricular geometry, heart rate, or blood pressure [5].

The authors found that a higher Tei index is linked to the death of children receiving CRRT. This is the same as what Asami and his colleagues found in their study. They found that high Tei index was associated with increased risk of all-cause mortality after aortic valve replacement [15].

Increased Tei index was found to be independent of fraction shortening and ejection fraction, which is consistent with other previous studies [16, 17].

In the present study authors found that EF and EF Z scores were not significantly different between survivors and non-survivors which is similar finding in study done by Abdel-Hady and his colleagues that found no significant difference regarding LVFS between septic neonates and their controls [18].

The authors noticed that patients with no pericardial effusion had statistically considerably better outcomes, which is consistent with previous research that indicated pericardial effusion is significantly linked to mortality [19].

The authors measured the mean pulmonary artery pressure and it was 29.89 ± 7.9 mmHg which is considered pulmonary hypertension according to pediatric references [20]. Approximately half of present patients had CKD that is a higher risk of pulmonary artery disease [21].

In this study, there was a considerable difference in the need for inotropic therapy between patients with good and unfavorable prognoses in concordance to prior investigations [12, 22].

The authors found that survivors started CRRT earlier than non-survivors, which is consistent with findings of Cortina and colleagues who concluded that mortality rate increased by 1% for every hour of delay after ICU admission [2].

Early initiation of CRRT is expected to improve fluid balance, electrolyte disruption, acid-base homeostasis, uremia, and toxin clearance more effectively than late therapy [23]. On the other hand, it raises the risk of vascular access complications, bleeding associated with anticoagulant use, abrupt changes in electrolyte levels and unnecessary medication clearance [24]. All these consequences make the decision for early initiation of CRRT challenging.

PRISM III score, which may not adequately account for AKI, is still the most widely used mortality risk prediction score [25]. Patients with higher scores were more prone to death in this study (*P* = 0.001), which is consistent with the findings of Jhang et al. [26].

Most of the present patients had elevated CRP levels before CRRT, although there was no significant difference in CRP levels before and after CRRT (*p* = 0.17), despite the fact that CRP has a low molecular weight (22–25 kDa), which is below the cut-off permeability limits of CRRT dialysis membranes [27]. CRP levels decreased non-significantly following CRRT due to delayed CRP testing.

CRP was considerably lower in survivors than in non-survivors (*p* = 0.03). This is consistent with other studies that linked high CRP levels to an increased risk of death [28, 29].

The present patients who required mechanical ventilation had a higher mortality rate than those who did not, as reported in prior studies [25]. This can be explained by most patients requiring mechanical ventilation had multi-organ failure, which elevated their risk of death.

In conclusion, higher Tei index, higher PRISM III score, higher CRP, an increased VIS in patients receiving CRRT, as well as respiratory failure at the time of CRRT commencement, were all related to higher mortality. The Tei index may be a predictor of poor outcome in children receiving CRRT.

This study has been limited by the type of patients as about half of them had CKD, which may be associated with cardiac dysfunction and may be an independent risk factor for a poor outcome.

## References

[1] Buccione E, Guzzi F, Colosimo D, et al. Continuous renal replacement therapy in critically ill children in the pediatric intensive care unit: a retrospective analysis of real-life prescriptions, Complications, and outcomes. Front Pediatr. 2021;9:696798. 34195164 10.3389/fped.2021.696798 PMC8236631

[2] Cortina G, McRae R, Hoq M, et al. Mortality of critically ill children requiring continuous renal replacement therapy: effect of fluid overload, underlying Disease, and timing of initiation. Pediatr Crit Care Med. 2019;20:314–22. 30431556 10.1097/PCC.0000000000001806

[3] Mirza S, Malik L, Ahmed J, et al. Accuracy of pediatric risk of mortality (PRISM) III score in predicting mortality outcomes in a pediatric intensive care unit in Karachi. Cureus. 2020;12:e7489. 32368422 10.7759/cureus.7489 PMC7193246

[4] Kompotiatis P, Wiley BM, Jentzer JC, Kashani KB. Echocardiographic parameters of patients in the intensive care unit undergoing continuous renal replacement therapy. PLoS ONE. 2019;14:e0209994. 30633756 10.1371/journal.pone.0209994 PMC6329514

[5] Ardahanli I, Akhan O, Sahin E, Akgun O, Gurbanov R. Myocardial performance index increases at long-term follow-up in patients with mild to moderate COVID-19. Echocardiography. 2022;39:620–5. 35294060 10.1111/echo.15340 PMC9111876

[6] Tissot C, Singh Y, Sekarski N. Echocardiographic evaluation of ventricular function—for the neonatologist and pediatric intensivist. Front Pediatr. 2018;6:79. 29670871 10.3389/fped.2018.00079 PMC5893826

[7] Dallaire F, Slorach C, Hui W, et al. Reference values for pulse wave Doppler and tissue doppler imaging in pediatric echocardiography. Circ Cardiovasc Imaging. 2015;8:e002167. 25632029 10.1161/CIRCIMAGING.114.002167

[8] Pollack MM, Patel KM, Ruttimann UE. PRISM III: an updated pediatric risk of mortality score. Crit Care Med. 1996;24:743–52. 8706448 10.1097/00003246-199605000-00004

[9] Gaies MG, Gurney JG, Yen AH, et al. Vasoactive–inotropic score as a predictor of morbidity and mortality in infants after cardiopulmonary bypass. Pediatr Crit Care Med. 2010;11:234–8. 19794327 10.1097/PCC.0b013e3181b806fc

[10] Chan YH. Biostatistics 104: correlational analysis. Singapore Med J. 2003;44:614–9. 14770254

[11] Ning B, Ye S, Lyu Y, Yin F, Chen Z. Effect of high-volume hemofiltration on children with sepsis. Transl Pediatr. 2020;9:101–7. 32477909 10.21037/tp.2020.03.13 PMC7237970

[12] Lee H-J, Son Y-J. Factors associated with in-hospital mortality after continuous renal replacement therapy for critically ill patients: a systematic review and meta-analysis. Int J Environ Res Public Health. 2020;17:8781. 33256008 10.3390/ijerph17238781 PMC7730748

[13] Singh Y. Echocardiographic evaluation of hemodynamics in neonates and children. Front Pediatr. 2017;5:201. 28966921 10.3389/fped.2017.00201 PMC5605552

[14] Basu S, Kim EJ, Sharron MP, et al. Strain echocardiography and myocardial dysfunction in critically ill children with multisystem inflammatory syndrome unrecognized by conventional echocardiography: a retrospective cohort analysis. Pediatr Crit Care Med. 2022;23:e145–52. 34636357 10.1097/PCC.0000000000002850 PMC8887681

[15] Asami M, Pilgrim T, Lanz J, et al. Prognostic relevance of left ventricular myocardial performance after transcatheter aortic valve replacement. Circ Cardiovasc Interv. 2019;12:e006612. 30626203 10.1161/CIRCINTERVENTIONS.118.006612

[16] Grignola JC, Ginés F, Guzzo D. Comparison of the Tei index with invasive measurements of right ventricular function. Int J Cardiol. 2006;113:25–33. 16325940 10.1016/j.ijcard.2005.10.012

[17] Demirkol S, Ozturk C, Balta S, Unlu M, Arslan Z. Subclinical left ventricular dysfunction in patients with obstructive sleep apnea. Med Princ Pract. 2015;25:299–300. 26624021 10.1159/000442881 PMC5588371

[18] Abdel-Hady HE, Matter MK, El-Arman MM. Myocardial dysfunction in neonatal sepsis: a tissue doppler imaging study. Pediatr Crit Care Med. 2012;13:318–23. 21725277 10.1097/PCC.0b013e3182257b6b

[19] Dalby ST, Tang X, Daily JA, Sukumaran S, Collins RT, Bolin EH. Effect of pericardial effusion on outcomes in children admitted with systemic Lupus Erythematosus: a multicenter retrospective cohort study from the United States. Lupus. 2019;28:389–95. 30744520 10.1177/0961203319828523

[20] Simonneau G, Montani D, Celermajer DS, et al. Haemodynamic definitions and updated clinical classification of pulmonary Hypertension. Eur Respir J. 2019;53:1801913. 30545968 10.1183/13993003.01913-2018 PMC6351336

[21] Bolignano D, Rastelli S, Agarwal R, et al. Pulmonary Hypertension in CKD. Am J Kidney Dis. 2013;61:612–22. 23164943 10.1053/j.ajkd.2012.07.029

[22] Kee YK, Kim D, Kim S-J, et al. Factors associated with early mortality in critically ill patients following the initiation of continuous renal replacement therapy. J Clin Med. 2018;7:334. 30297660 10.3390/jcm7100334 PMC6210947

[23] Andres-Hernando A, Altmann C, Bhargava R, et al. Prolonged acute kidney injury exacerbates lung inflammation at 7 days post-acute kidney injury. Physiol Rep. 2014;2:e12084. 25052489 10.14814/phy2.12084 PMC4187574

[24] Karakala N, Tolwani AJ. Timing of renal replacement therapy for acute kidney injury. J Intensive Care Med. 2019;34:94–103. 29739260 10.1177/0885066618774257

[25] Boschee ED, Cave DA, Garros D, et al. Indications and outcomes in children receiving renal replacement therapy in pediatric intensive care. J Crit Care. 2014;29:37–42. 24246752 10.1016/j.jcrc.2013.09.008

[26] Jhang WK, Kim YA, Ha EJ, et al. Extrarenal sequential organ failure assessment score as an outcome predictor of critically ill children on continuous renal replacement therapy. Pediatr Nephrol. 2014;29:1089–95. 24469438 10.1007/s00467-013-2741-z

[27] Honore PM, Jacobs R, Hendrickx I, De Waele E, Gorp VV, Spapen HD. Biomarking’ Infection during continuous renal replacement therapy: still relevant ? Crit Care. 2015;19:232. 26002320 10.1186/s13054-015-0948-z PMC4488982

[28] Aygun F. Procalcitonin value is an early prognostic factor related to mortality in admission to pediatric intensive care unit. Crit Care Res Pract. 2018;2018:9238947. 30675399 10.1155/2018/9238947 PMC6323529

[29] Qu R, Hu L, Ling Y, et al. C-reactive protein concentration as a risk predictor of mortality in intensive care unit: a multicenter, prospective, observational study. BMC Anesthesiol. 2020;20:292. 33225902 10.1186/s12871-020-01207-3 PMC7680994

